# Physiological responses of rosewoods *Dalbergia cochinchinensis* and *D. oliveri* under drought and heat stresses

**DOI:** 10.1002/ece3.6744

**Published:** 2020-09-09

**Authors:** Tin Hang Hung, Rosemary Gooda, Gabriele Rizzuto, Thea So, Bansa Thammavong, Hoa Thi Tran, Riina Jalonen, David H. Boshier, John J. MacKay

**Affiliations:** ^1^ Department of Plant Sciences University of Oxford Oxford UK; ^2^ Institute of Forest and Wildlife Research and Development Phnom Penh Cambodia; ^3^ National Agriculture and Forestry Research Institute Forestry Research Center Vientiane Lao PDR; ^4^ Forest Genetics and Conservation Center for Biodiversity and Biosafety Institute of Agricultural Genetics Vietnam Academy of Agricultural Sciences Hanoi Vietnam; ^5^ Bioversity International, Malaysia Office c/o TNCPI, University Putra Malaysia, off Lebuh Silikon Serdang Malaysia

**Keywords:** drought tolerance, ecophysiology, heat shock, rosewoods, stress response

## Abstract

*Dalbergia cochinchinensis* and *D. oliveri* are classified as vulnerable and endangered, respectively, in the IUCN Red List and under continued threat from deforestation and illegal harvesting for rosewood. Despite emerging efforts to conserve and restore these species, little is known of their responses to drought and heat stress, which are expected to increase in the Greater Mekong Subregion where the species co‐occur and are endemic. In this study of isolated and combined drought and heat effects, we found that *D. oliveri* had an earlier stomatal closure and more constant midday water potential in response to increasing drought level, suggesting that *D. oliveri* is relatively isohydric while *D. cochinchinensis* is relatively anisohydric. Heat shock and drought had synergistic effects on stomatal closure. Our results indicate contrasting relationships in water relations, photosynthetic pigment levels, and total soluble sugars. An increase in chlorophyll a was observed in *D. cochinchinensis* during drought, and a concomitant increase in carotenoid content likely afforded protection against photo‐oxidation. These physiological changes correlated with higher total soluble sugars in *D. cochinchinensis*. By contrast, *D. oliveri* avoided drought by reducing chlorophyll content and compromising productivity. Anisohydry and drought tolerance in *D. cochinchinensis* are adaptations which fit well with its ecological niche as a pioneering species with faster growth in young trees. We believe this understanding of the stress responses of both species will be crucial to their effective regeneration and conservation in degraded habitats and in the face of climate change.

## Graphical abstract

Endangered rosewood species *Dalbergia cochinchinensis* and *D. oliveri* demonstrated contrasting hydraulic and photosynthetic responses under drought and heat stress. Anisohydry and drought tolerance in *D. cochinchinensis* are adaptations which fit well with its ecological niche as a pioneering species. We believe understanding of the stress responses of both species will be crucial to their effective regeneration and conservation in degraded habitats and in the face of climate change.

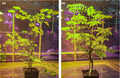

## 1 INTRODUCTION

**FIGURE 1:**
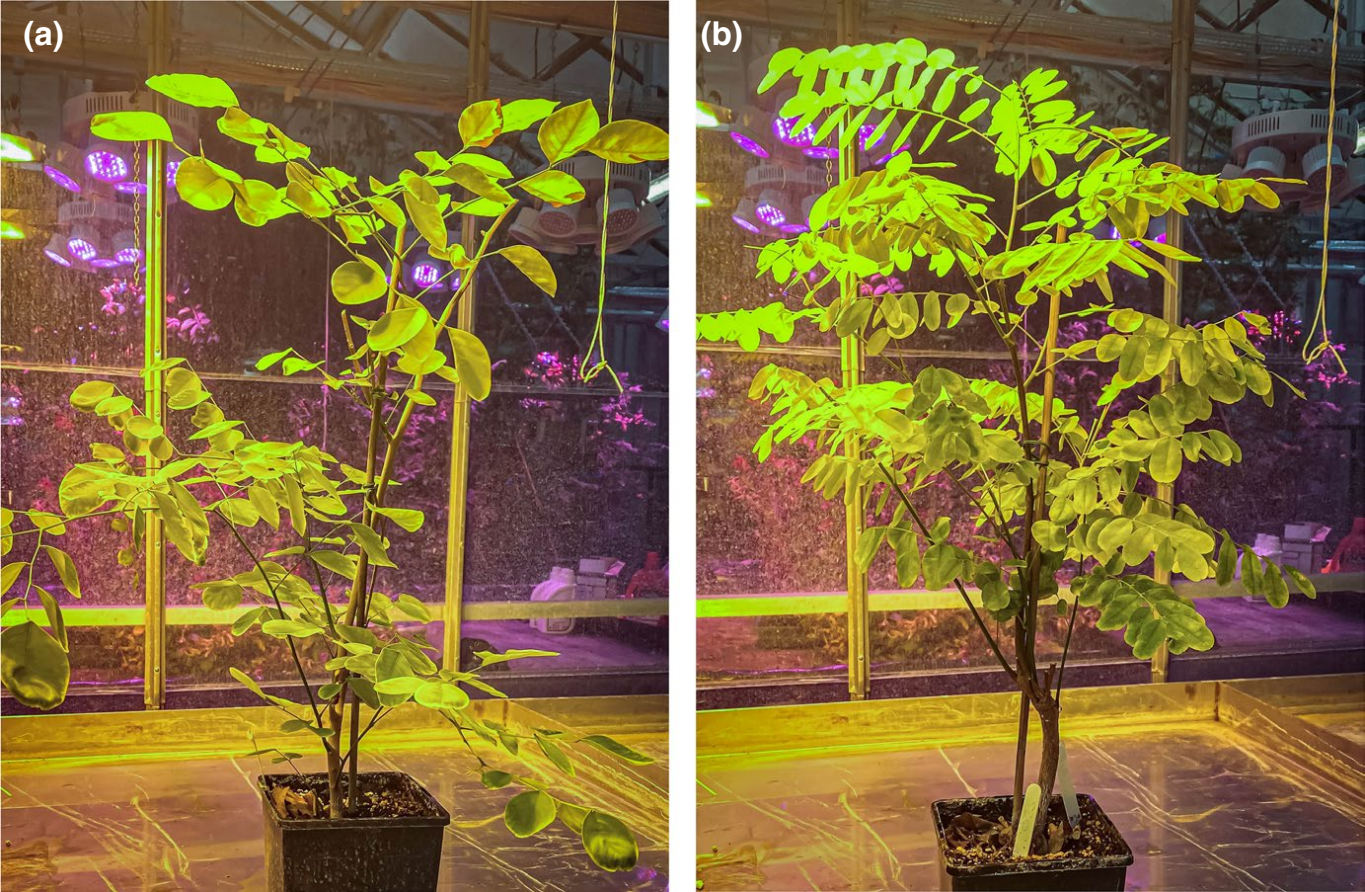
Photograph of (a) *Dalbergia cochinchinensis* and (b) *D. oliveri* in a greenhouse (not taken during this study)

The pantropical genus *Dalbergia* Linn. f. (Fabaceae: Faboideae) contains around 250 species (Vatanparast, 2013), many of which produce valuable heartwood timber known as rosewood (Winfield, Scott, & Graysn, 2016), which is used to manufacture luxury furniture, boats, and musical instruments (Bhagwat, Dholakia, Kadoo, Balasundaran, & Gupta, 2015). Growing demand and diminishing supply have drastically increased the economic value of rosewood, resulting in much illegal harvesting and poorly regulated exploitation of natural populations. Among these are *Dalbergia cochinchinensis* Pierre and *D. oliveri* Gamble ex Prain (Figure 1), both of which are endemic to Cambodia, Laos, Thailand, and Vietnam within the Greater Mekong Subregion (GMS). *D. cochinchinensis* was once the most sought‐after rosewood species globally, but it is now virtually commercially extinct, as is *D. oliveri* (EIA, 2017). They were classified as vulnerable and endangered, respectively, in the IUCN Red List in 1998, with international trade strictly regulated since 2017 under CITES Appendix II.

Responses to their declining populations have seen new initiatives in the Greater Mekong Subregion to conserve and restore both species, such as through improving capacity for seed collection, germplasm supply, and propagation (APFORGEN, 2018; CDRI, 2014; Luoma‐aho, Hong, Ramanatha Rao, & Sim, 2003). In addition, some *Dalbergia* species are deemed suitable candidates for use in forest landscape restoration in the region (Aerts, 2009), where intense land conversion has degraded sites (Tanaka, 2008). Successful incorporation of valuable and endangered species like *Dalbergia* in forest restoration programs can achieve both conservation and restoration goals (Kaewkrom, Gajaseni, Jordan, & Gajaseni, 2005; Sakai, 2009). A sustainable supply of diverse germplasm is essential for the success of such projects (Maningo & Thea). Recently, the importance of investigating the response to climatic stresses of germplasm used in forest restoration and other tree planting has been recognized (2017), since many forest restoration projects around the world have neglected the adaptability of seeds in the planting sites (Jalonen, Valette, Boshier, Duminil, & Thomas, 2018). For reforestation to succeed, it is crucial to know about the ranges of abiotic conditions that a species can tolerate, allowing their planting in appropriate regions. There is particular concern for the effect of climate change within the Greater Mekong Subregion with evidence pointing to increased temperature, variability in runoff, and prolonged agricultural droughts (MRC, 2010). Simultaneously, water availability may decrease in the region due to increasing urbanization and changes in river infrastructure such as upstream dams in China (Hughes, 2017). However, we have little understanding of the physiological adaptations of *D. cochinchinensis* and *D. oliveri*, which hinders informed decision‐making in both conservation and forest restoration initiatives.

Imminent effects of climate change will, in many areas, result in rapid increases in temperatures, drought, and extreme weather (Li, 2015). The sessile habit and longevity of tree species mean they must have sufficient phenotypic plasticity to tolerate the wide range of environmental conditions that occur during their lifespans (Estravis‐Barcala, 2019). Similarly, trees are potentially more vulnerable than other plants under changing climate scenarios as they have long generation times, with the persistence of a particular tree species dependent on adaptive capacity to stress, plasticity, and migration potential (Aitken, Yeaman, Holliday, Wang, & Curtis‐McLane, 2008). Extreme abiotic conditions caused by climate change will potentially be detrimental to primary productivity, ecological functions, and associated biodiversity of these forests (Sitch, 2008). Vulnerability of forests to tree die‐off due to climate change has become a focus of forest sustainability (Allen, Breshears, & McDowell, 2015), with extensive tree climate‐induced mortality well‐documented worldwide (Allen, 2010; McDowell, 2008; Pollastrini, Puletti, Selvi, Iacopetti, & Bussotti, 2019). In particular, seedling recruitment and survival are considered critical bottlenecks in tree life history and have an important role in shifts in species’ range under changing climate (Canham & Murphy, 2016). Migration to adapted ecological niches and acclimation in an existing range are important mechanisms for tree survival in a changing climate (Brodribb, Powers, Cochard, & Choat, 2020).

Although knowledge of the ability of tree species to tolerate environmental stresses is essential for our understanding of how trees will respond to the effects of climate change (Estravis‐Barcala, 2019), we lack data on the stress tolerance of specific taxa (Chaves, 2002). Water availability varies spatially and temporally and has also been found to be an important determinant of functional traits in trees (Terra, 2018). Trees have evolved strategies to balance hydraulic conductance and resource allocation in response to water deficit. One of these strategies is establishing barriers for evaporation to achieve homeostasis of tissue water status (Estravis‐Barcala, 2019). Other strategies exist to maintain their metabolism at a lower water potential (Polle, Chen, Eckert, & Harfouche, 2019) through osmotic adjustment and protection from photo‐oxidation (Pintó‐Marijuan & Munné‐Bosch, 2014). However, prolonged drought stress that exceeds the drought resistance threshold can lead to mortality associated with hydraulic failure, carbon starvation, and the demography of biotic agents (McDowell, 2008).

Temperature also plays a major role in determining the distribution of tree species, as it significantly regulates tree growth and development. Trees can usually utilize transpiration to allow water evaporation for temperature regulation (Urban, Ingwers, McGuire, & Teskey, 2017), but excessive heat stress is an important driver of tree die‐off in natural ecosystems (McDowell, 2018). Studies have shown that heat stress impairs photosystems, stimulates photorespiration, and encourages production of volatile compounds (Li, 2014; Rizhsky, Liang, & Mittler, 2002). Extreme heat reduces tree vigor, fecundity, and growth, significantly affecting survival (Teskey, 2015).

Different stresses often occur simultaneously in the field but there are few reports on interactions between such stresses in trees (Chaves, 2002). It has been proposed that drought and heat stresses are intrinsically linked and produce positive feedbacks to intensify their effects (Stéfanon, Drobinski, D'Andrea, Lebeaupin‐Brossier, & Bastin, 2014), but new evidence indicates that combinations of stresses can invoke responses that are distinct from those of the individual stresses (Zandalinas, Mittler, Balfagón, Arbona, & Gómez‐Cadenas, 2018). For example, the combination of heat and drought stress could reduce the negative effect of drought stress by preserving predawn water potential and malondialdehyde (Correia, 2018). These interactions and responses are species‐specific and are expected to be complex and difficult to predict, as response mechanisms vary between stresses (Sheel, Göran, Löfvenius, & Marie‐Charlotte, 2013).

The objective of the present study is to develop an understanding of the physiological responses of 3‐month‐old *D. cochinchinensis* and *D. oliveri* seedlings under controlled conditions of heat and drought stress. First, we compare and characterize the hydraulic responses in the two species when exposed to drought. Second, we determine the effects of isolated and combined stresses of heat and drought on hydraulic, leaf, and photosynthetic traits. We discuss the findings in relation to life‐history traits and ecological niches of these species and formulate implications for their conservation and use in restoration.

## 2 METHODS

### 2.1 Plant materials

Dried seeds of *D. cochinchinensis* and *D. oliveri* were provided by the Forest Research Center, Lao PDR, and the Institute of Forest & Wildlife Research & Development, Cambodia, respectively, in 2018. We scarified the seeds by placing them in 70°C distilled water, which was then left to cool to room temperature overnight and germinated them on 1% agar in a plant growth cabinet MLR‐350 (Sanyo, Watford, United Kingdom) at 25°C and photoperiod 12L/12D. Germinants were transferred to 1‐L pots in a soil‐perlite 3:1 (v:v) mixture in a greenhouse set to 30°C, 80% RH, and 12L/12D. 128 plants were kept in trays of 8 pots and randomized with equal numbers of individuals of *D. cochinchinensis* and *D. oliveri*. Plants were watered to maintain at substrate capacity and fertilized once a week using N‐P‐K 20:20:20 fertilizer (Chempak, Suffolk, United Kingdom).

### 2.2 Experimental design

**FIGURE 2:**
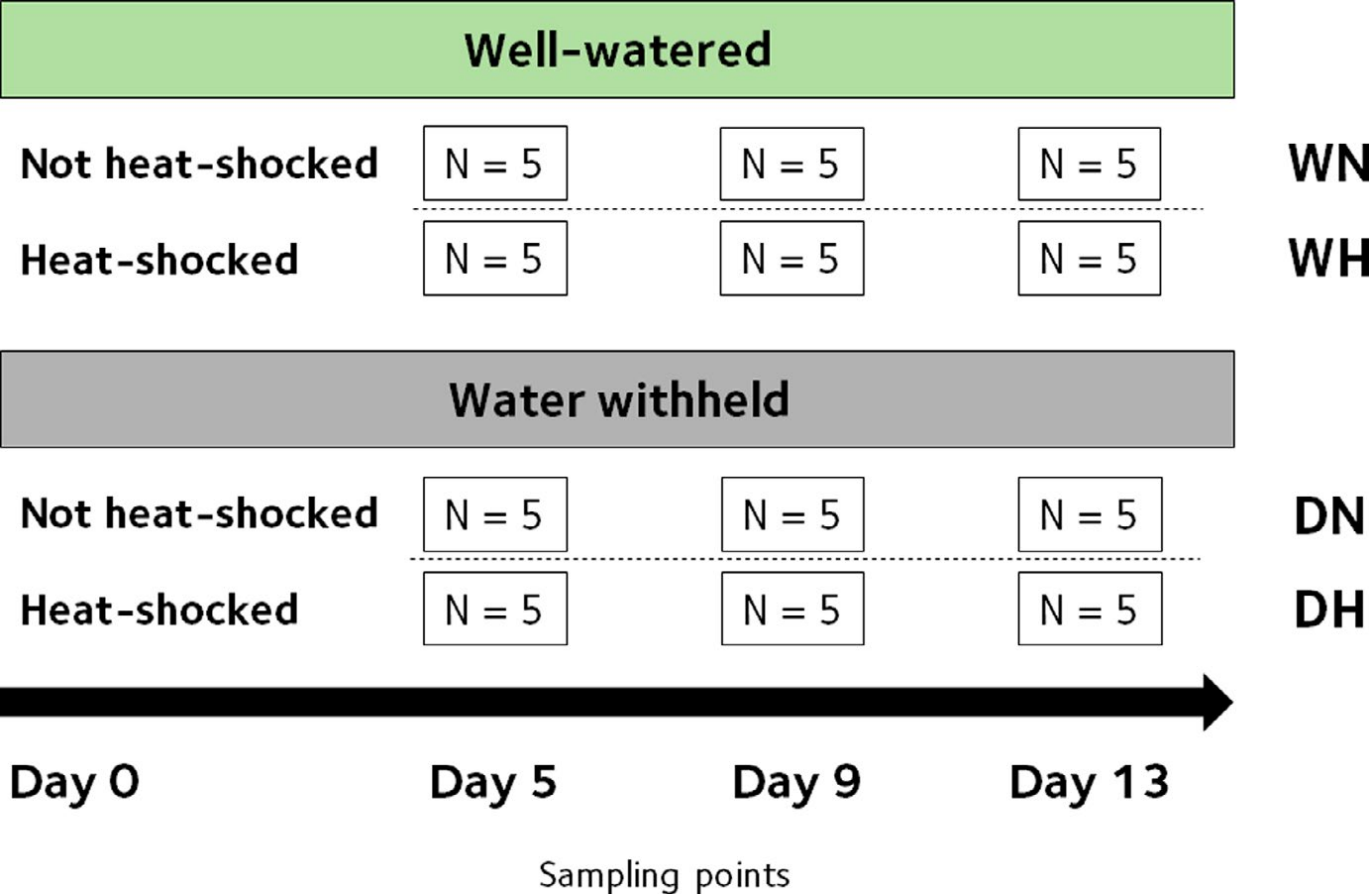
Conceptual diagram of the experimental design in this study

After 3 months of growth in the greenhouse, the experiment began on the 12 June 2019 (Day 0), on which all plants were watered. The design included a total of 4 treatments: drought treatment (D), well‐watered control (W), heat shock treatment (H), or non‐heat shock control (N) (Figure 2). Each of the trays of 8 plants was randomly assigned as either D or W, while each plant of the trays was then randomly assigned to H or N. This resulted in each individual being assigned to one of four treatment combinations: drought and heat shock (DH), well‐watered and heat shock (WH), drought and non‐heat shock (DN), and well‐watered and non‐heat shock (WN). The well‐watered plants were watered every other day to maintain them at substrate capacity and all water was withheld from the drought plants. Each individual plant was randomly assigned to one of the three sampling points: 5, 9, and 13 days from the beginning of treatments according to Figure 2, each five biological replicates were present for each sampling group.

For the heat shock treatment, we placed half of the individuals to be sampled at a given time point in a plant growth cabinet MLR‐350 (Sanyo, Watford, United Kingdom) at 38°C for 4 hr from 08:00 to 12:00 before sampling. Five biological replicates were finally present for each sampling group.The samples were made up of homogeneous leaves that were snap‐frozen in liquid nitrogen and kept at –80°C for subsequent analyses. Fully expanded branches were also cut at the nodes and kept in cooler bags for water potential, mass, and area measurements.

### 2.3 Water relation measurements

Soil water content (SWC) was measured daily using a ML3 ThetaProbe Soil Moisture Sensor (Delta‐T Devices Ltd. Cambridge, England) by placing the sensor probe in the soil at the center of the pot. Stomatal conductance (*g*_*s*_) was determined daily using an SC‐1 leaf porometer (METER Group, Inc.) to measure vapor flux through the stomata on fully expanded leaves for 30 s. Midday water potential (Ψ_MD_) was measured by using a scholander‐type pressure chamber SKPM140 (Skye Instruments Ltd.) on a fully expanded branch. Pressure was increased inside the chamber with compressed nitrogen, until moisture appeared on the cut end and the reading recorded.

### 2.4 Leaf dry matter content and specific leaf area

An office scanner was used to obtain electronic images of leaves on a fully expanded branch from each plant. The program ImageJ 1.52s (Schneider, Rasband, & Eliceiri, 2012) was then used to measure the total leaf area of a fully expanded branch for each individual. These leaves were then dried in an oven at 65°C until the weight reading remained constant for two consecutive days.

Leaf dry matter content (LDMC) and specific leaf area (SLA) were deduced using the following equations (Pérez‐Harguindeguy, 2013):

(1) $$\text{Leaf dry matter content}=\frac{\text{Oven-dried mass of leaf}\;(\text{mg})}{\text{Fresh mass of leaf}\;(\text{mg})}$$

(2) $$\text{Specific leaf area}\;({\text{cm}}^{2}/\text{mg})=\frac{\text{Area of leaf}\;({\text{cm}}^{2})}{\text{Oven-dried mass of leaf}\;(\text{mg})}$$

### 2.5 Pigment quantification

Photosynthetic pigments were extracted using cold acetone‐50 mM Tris buffer pH 7.8 (80:20 v:v) following Sims & Gamon's protocols (Sims & Gamon, 2002). Absorbances of the extract were read at 470, 537, 647, and 663 nm using Helios Gamma UV‐Vis Spectrophotometer (Thermo Fisher Scientific). Concentrations of anthocyanin (*Ac*), chlorophyll a (*Chl_a_*), b (*Chl_b_*), and carotenoids were determined using Sims and Gamon's formulae:

(3) $$Ac\;\left(\mu \text{mol}/\text{ml}\right)=0.08173A_{537}‐0.00697A_{647}‐0.002228A_{663}$$

(4) $$Chl_{a}\;\left(\mu \text{mol/ml}\right)=0.01373A_{663}‐0.000897A_{537}‐0.003046A_{647}$$

(5) $$Chl_{b}\;\left(\mu \text{mol/ml}\right)=0.02405A_{647}‐0.004305A_{537}‐0.005507A_{663}$$

(6) $$\text{Carotenoids}\;\left(\mu \text{mol/ml}\right)=(A_{470}‐\left(17.1\times \left(Chl_{a}+Chl_{b}\right)‐9.479Ac\right))\times 119.26$$

### 2.6 Total soluble sugars quantification

Total soluble sugars (TSS) were extracted from 50 mg of frozen leaves in 80% (v/v) ethanol for 1 hr at 80°C. TSS concentration was determined by Osaki's anthrone method (Osaki, Shinano, & Tadano, 1991), in which the extract was mixed with anthrone and sulfuric acid. After heating the mixture at 100°C for exactly 10 min, absorbance was read at 625 nm using Helios Gamma UV‐Vis Spectrophotometer (Thermo Fisher Scientific). The concentration was determined according to a D‐glucose standard curve.

### 2.7 Statistical analysis

The experiment used a split‐plot design: The drought treatment was randomized at the tray level (main‐plot), while heat shock was randomized at the individual plant level (split‐plot). We log‐transformed the *g*_*s*_, SLA, *Ac* *, Chl_b_*, and carotenoids content to correct for normality.

To compare and characterize the hydraulic responses in the two species over time, we applied linear‐plateau regression of *g_s_* against SWC in the drought treatment groups for both species. Best‐fit estimates of the intercept (*c*), the slope (*m*), and the critical value of SWC (*x'*) were predicted using nonlinear least‐squares method (Bates & Watts, 1988) with the following equation:

(7) $$g_{s}=\left\{\begin{array}{c} mx+c,\;\text{if}\;x<x^{\prime }\\ mx^{\prime }+c,\;\text{if}\;x\geq x^{\prime } \end{array}\right)$$

To test the effects of isolated and combined stresses on the hydraulic, leaf, and photosynthetic traits, we performed main‐plot analyses on data subsets which averaged the response variables, including *g_s_*, Ψ_MD_, LDMC, SLA, *Ac*, *Chl_a_*, *Chl_b_*, carotenoids, and TSS, over the combinations of block, species, and two treatments, and performed split‐plot analyses on the full dataset while controlling for block. The fixed effects of the two stresses and their interaction were tested with the following equation:

(8) $$\begin{array}{c} Y=\mu +{\text{drought}}_{a}+{\text{block}}_{i}+\eta_{\mathrm{ai}}+{\text{species}}_{b}+{\text{day}}_{c}+{\text{heatshock}}_{d}+\\ \;{\left(\text{drought}*\text{heatshock}*\text{day}*\text{species}\right)}_{\mathrm{abcd}}+\varepsilon_{\mathrm{abcdi}} \end{array}$$

*Y*: response variable; *μ*: mean; drought*_a_*: fixed effect of drought treatment; block*_i_*: fixed effect of block; *η_a__i_*: whole‐plot error; species*_b_*: fixed effect of species; day*_c_*: fixed effect of number of days since water withholding; heatshock*_d_*: fixed effect of heat shock treatment; (drought*heatshock*day*species)*_abcd_*: interaction between explanatory variables; *ε_abcdi_*: split‐plot error.

We used R 3.6.2 to perform the randomization for experimental design, statistical analysis, and data visualization. An analysis of variance (ANOVA) table was computed for the statistical model of each response variable.

## 3 RESULTS

The plants were divided into drought (D) and well‐watered control (W) treatment groups following a split‐plot experimental design. Physiological traits were measured after 5, 9, and 13 days of treatment. On the sampling days only, we also applied a heat shock (H) treatment to half of the individuals in each sampling group with the other not exposed to heat (N). Hence, there were four treatment groups: DH, DN, WH, WN (Figure 2).

### 3.1 Stomatal conductance

We found a statistically significant difference in slopes of stomatal conductance (*g_s_*) (*p* = .0134; Table S1) between the drought and well‐watered (control) treatments but not in intercepts (*p* = .2308; Table S2). This observation indicated that water relations were similar among the two groups at the start of the experiment and that *g_s_* decreased with the drought treatment in both species.

**FIGURE 3:**
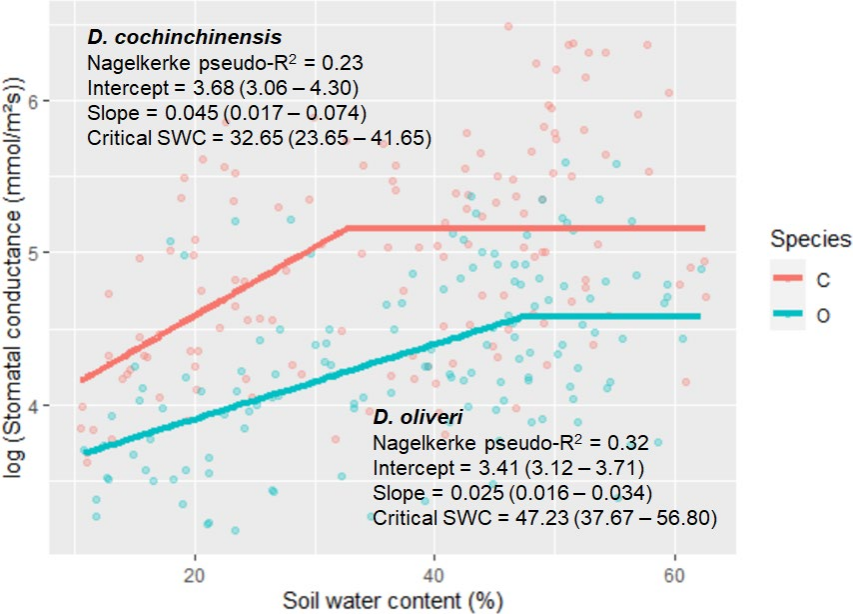
Linear‐plateau regression of stomatal conductance (*g_s_*) against soil water content (SWC) of *Dalbergia cochinchinensis* (red) and *D. oliveri* (blue). Goodness of fit was tested using Nagelkerke method. Values in the brackets represent 95% confidence interval

We employed a linear‐plateau nonlinear least‐squares fit to model the relationship between *g_s_* and soil water content (SWC) and searched for a critical value of SWC that affects the stomatal aperture (Figure 3). We found that the critical SWC of *D. cochinchinensis* (32.65%, *p* = 4.65e−11) was lower than *D. oliveri* (47.23%, *p* < 2e−16), suggesting that *D. cochinchinensis* closed its stomata later than *D. oliveri* in response to decreasing SWC.

**FIGURE 4:**
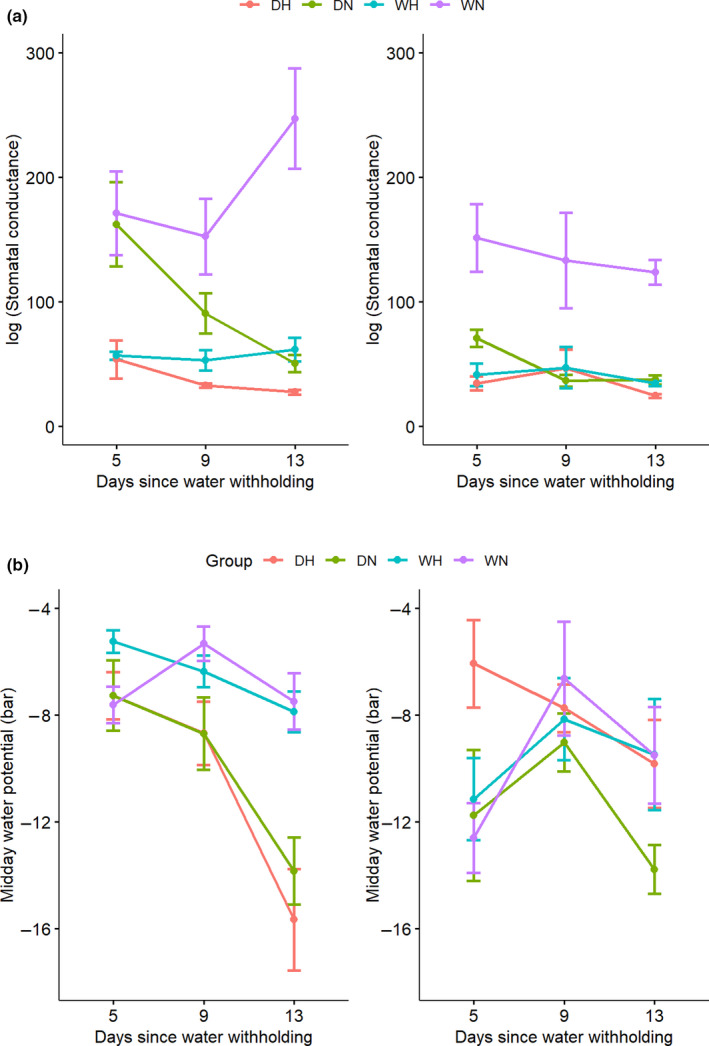
(a) Stomatal conductance (*g_s_*) and (b) midday branch water potential (Ψ_MD_) of *Dalbergia cochinchinensis* (left) and *D. oliveri* (right) at 5, 9, and 13 days from the beginning of drought treatment. Treatment groups were as follows: DH (drought and heat shock), DN (drought and non‐heat shock), WH (well‐watered and heat shock), and WN (well‐watered and non‐heat shock)

When analyzing all four treatment groups on sampling days, we obtained the same significant decrease in *g_s_* in drought treatment (*p* = 2.401e−5; Table S3 and Figure 4a). We found that *D. oliveri* had a lower *g*_*s*_ than *D. cochinchinensis* (*p* = 3.366e−06). In addition, we found a significant decrease in heat shock treatment (*p* = 2.936e−15) and a significant drought × heat interaction in *g_s_* (*p* = .000666). The interaction effect was the strongest on day 13: The *g_s_* of combined stress was lower (27.54 mmol/m^2^s) than either drought (50.48) or heat (61.8) alone in *D. cochinchinensis*. In *D. oliveri*, the *g_s_* of combined stress was also lower (24.56 mmol/m^2^s) than either drought (37.46) or heat (34.66) alone.

### 3.2 Midday water potential

We observed a significant decrease in midday water potential (Ψ_MD_) in the drought treatment (*p* = .03955; Table S4 and Figure 4b). A significant interaction was found between the drought treatment and species, (*p* = .004137), indicating differential responses and vulnerabilities, with *D. cochinchinensis* displaying a faster decrease in Ψ_MD_ over time than *D. oliveri*. The Ψ_MD_ of the *D. cochinchinensis* drought treatment group dropped drastically from –7.27 to –13.84 bar between day 5 and 13, while that for *D. oliveri* remained relatively stable from –11.75 to –13.78 bar.

### 3.3 Leaf dry matter content and specific leaf area

**FIGURE 5:**
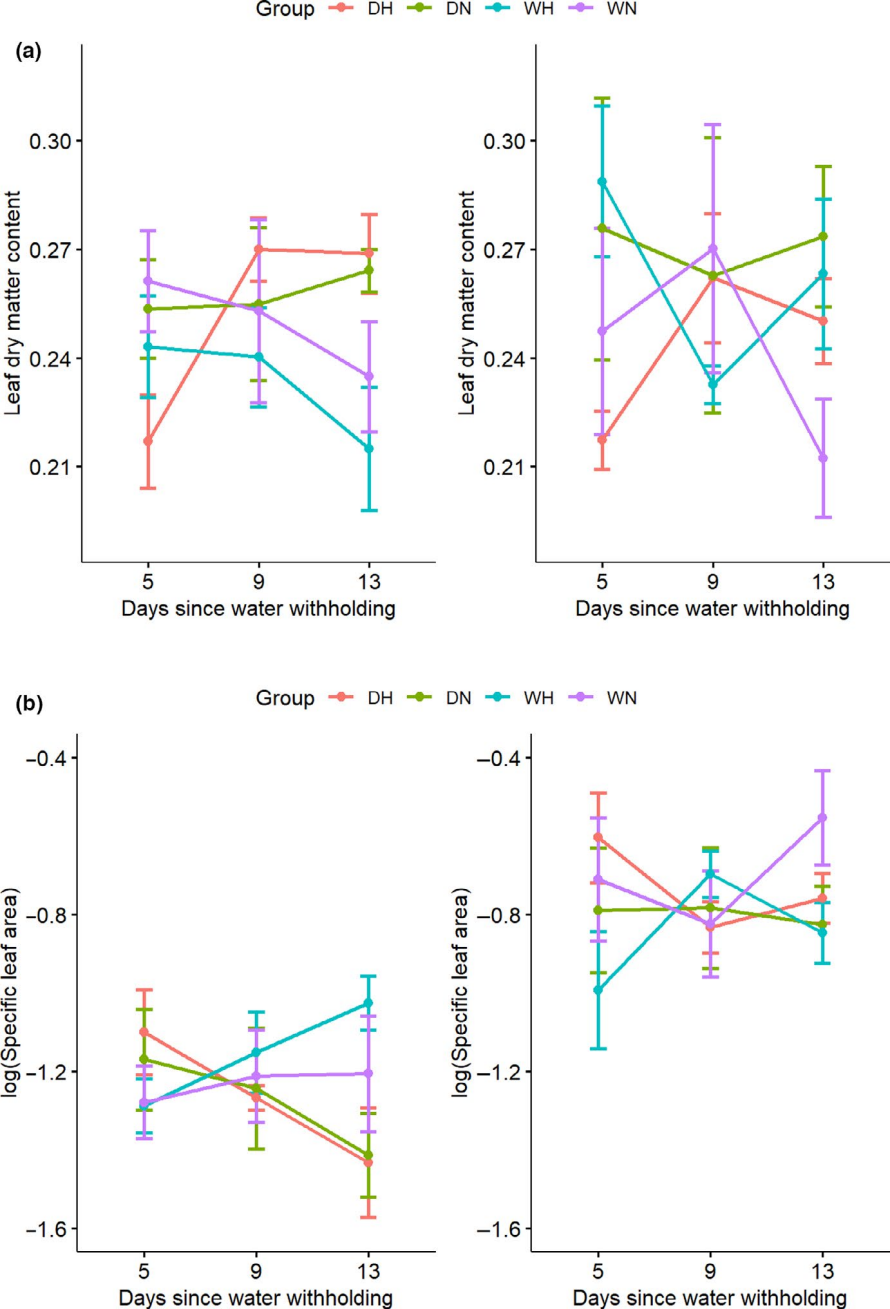
(a) Leaf dry matter content (LDMC) and (b) specific leaf area (SLA) of *Dalbergia cochinchinensis* (left) and *D. oliveri* (right) at 5, 9, and 13 days from the beginning of drought treatment. Treatment groups were as follows: DH (drought and heat shock), DN (drought and non‐heat shock), WH (well‐watered and heat shock), and WN (well‐watered and non‐heat shock)

We found no significant effect of the drought and heat treatments on leaf dry matter content (LDMC) and specific leaf area (SLA) (Table S5 and Figure 5a). However, SLA was significantly different between species (*p* = 1.061e−12; Table S6 and Figure 5b), with *D. cochinchinensis* having a lower SLA (0.30 cm^2^/mg) than *D. oliveri* (0.48 cm^2^/mg).

### 3.4 Pigments

**FIGURE 6:**
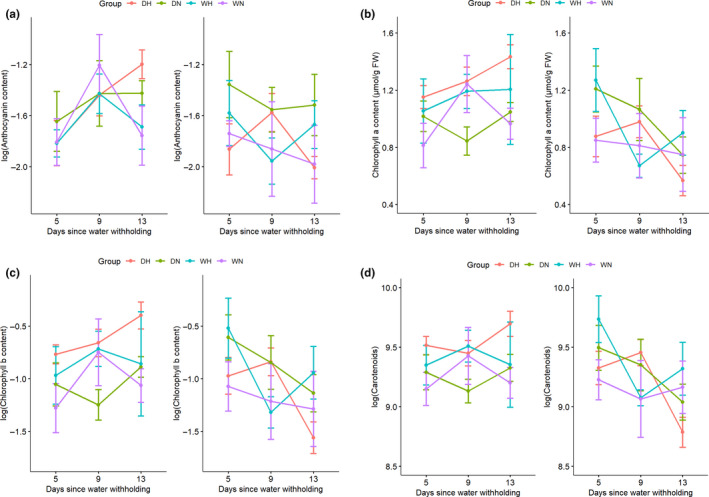
(a) Anthocyanin content, (b) chlorophyll a content (*Chl_a_*), (c) chlorophyll b content (*Chl_b_*), and (d) carotenoid content of *Dalbergia cochinchinensis* (left) and *D. oliveri* (right) at 5, 9, and 13 days from the beginning of drought treatment. Treatment groups were as follows: DH (drought and heat shock), DN (drought and non‐heat shock), WH (well‐watered and heat shock), and WN (well‐watered and non‐heat shock)

We observed no significant effect of either drought or heat shock stresses on anthocyanin content (Table S7 and Figure 6a). Chlorophyll a content (*Chl_a_*) was significantly affected by drought treatment (*p* = .02927; Table S8 and Figure 6b), species (*p* = .039284), and the interaction between both stresses and species (*p* = .034303). We found opposite trends of change in *Chl_a_* content between species, such that combined stresses increased *Chl_a_* from 1.15 to 1.43 μmol/g FW in *D. cochinchinensis* and decreased *Chl_a_* from 0.876 to 0.568 μmol/g FW in *D. oliveri*. The drought treatment seemed to amplify the difference between species, resulting in the highest *Chl_a_* in *D. cochinchinensis* and lowest in *D. oliveri*. We observed no significant effect of drought and heat shock stresses on chlorophyll *b* content (*Chl_b_*) (Table S9 and Figure 6c).

Carotenoid content was significantly affected by the drought treatment (*p* = .03966; Table S10 and Figure 6d) and the combination of drought, species, and days (*p* = .003470). An opposite trend between species was observed in carotenoids similar to *Chl_a_*, where carotenoid content increased over time following drought from 11.27 to 11.54 mmol/g FW in *D. cochinchinensis* but decreased from 14.27 to 8.63 mmol/g in *D. oliveri*.

### 3.5 Total soluble sugars

**FIGURE 7:**
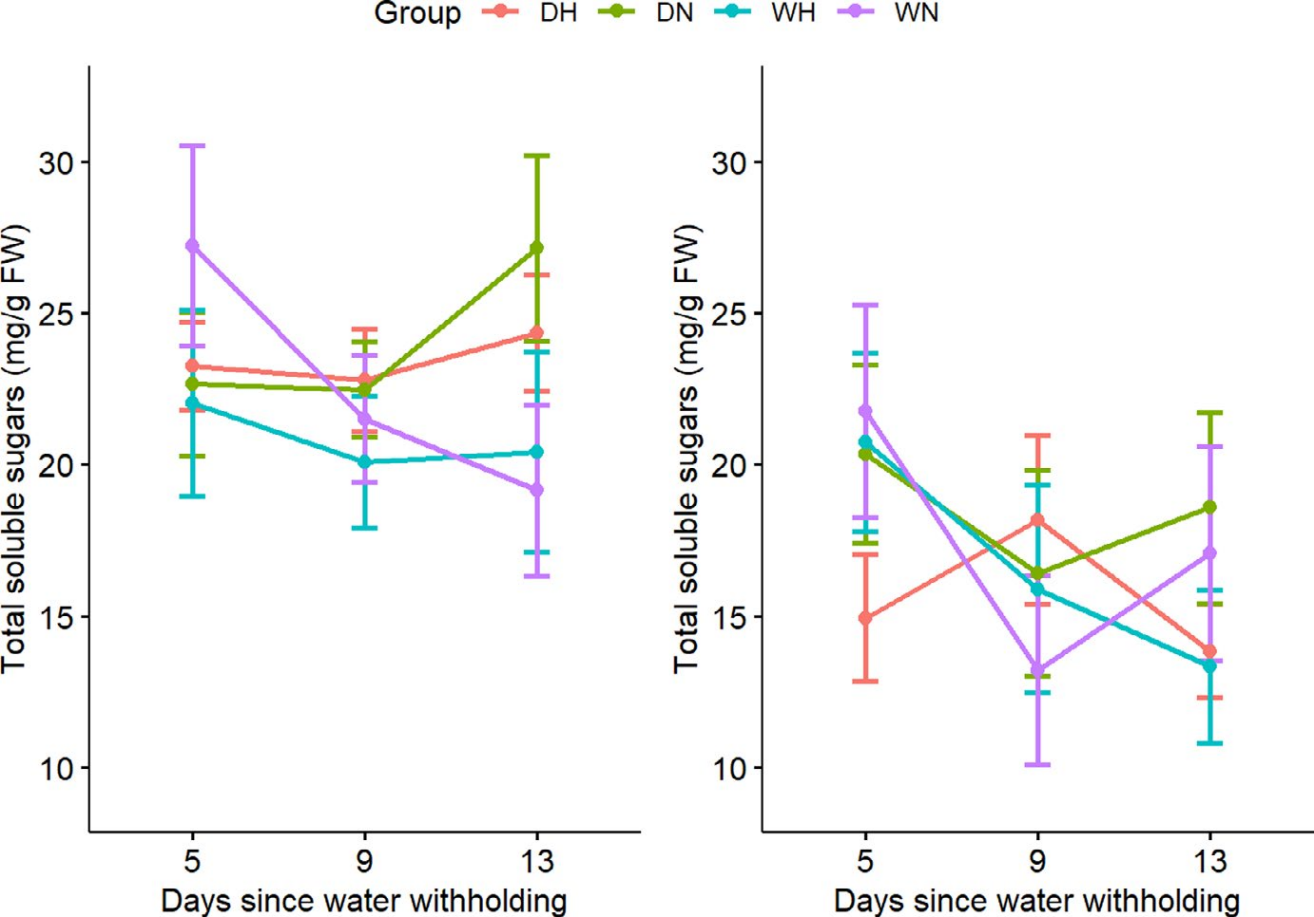
Total soluble sugars content (TSS) of *Dalbergia cochinchinensis* (left) and *D. oliveri* (right) at 5, 9, and 13 days from the beginning of drought treatment. Treatment groups were as follows: DH (drought and heat shock), DN (drought and non‐heat shock), WH (well‐watered and heat shock), and WN (well‐watered and non‐heat shock)

We found that the total soluble sugars content (TSS) changed significantly with the drought treatment (*p* = .03993; Table S11 and Figure 7) and increased slightly from 22.66 to 27.14 mg/g FW in *D. cochinchinensis*, but decreased slightly from 20.34 to 18.58 mg/g FW in *D. oliveri*. *D. cochinchinensis* maintained a higher TSS than *D. oliveri* (*p* = 2.586e−6).

## 4 DISCUSSION

We report short‐term physiological changes in seedlings of *D. cochinchinensis* and *D. oliveri* in response to isolated and combined stress treatments of drought and heat. Most of the responses are found to be induced by drought only but the two stresses have an additive effect on reducing stomatal conductance. These stress responses included several physiological factors and may highlight metabolic vulnerabilities, which follow from adaptations to environmental conditions. Our observations indicate changes in water relations, photosynthetic pigments, and soluble sugars. Our results suggest possible trade‐offs between hydraulic integrity and gas exchange associated with photosynthesis during drought, or between hydraulic integrity and temperature regulation during combined heat and drought stress. We discuss our findings as potential indicators of adaptation to different ecological niches for *D. cochinchinensis* and *D. oliveri* and the implications for their conservation and restoration.

### 4.1 Water relations

The observed differences in stomatal conductance (*g_s_*) and water potential (Ψ_MD_) over time reflected contrasting hydraulic responses in the two species: (1) stomatal closure occurred at a higher soil water content (or lower water deficit) in *D. oliveri* leaves than in *D. cochinchinensis*, and (2) Ψ_MD_ was relatively stable in *D. oliveri* until the last day of the drought treatment but decreased rapidly in *D. cochinchinensis*. The ability to maintain Ψ_MD_ and regression of *g_s_* in response to increasing water deficit is common differentiators of iso‐anisohydric behaviors (Hochberg, Rockwell, Holbrook, & Cochard, 2018): *D. oliveri* fit more closely with an isohydric response, characterized by earlier stomatal closure and thus maintaining a more constant Ψ_MD_ (Lavoie‐Lamoureux, Sacco, Risse, & Lovisolo, 2017). By contrast, *D. cochinchinensis* was more anisohydric, maintaining stomatal aperture until experiencing severe drought, when Ψ_MD_ decreases drastically (Bergel‐Landefeldt, 1936). However, recent research suggests that species naturally present a continuum of iso‐anisohydry, and very few species could be categorized in a strict dichotomy of iso‐anisohydry (Klein, 2014; Martínez‐Vilalta, Poyatos, Aguadé, Retana, & Mencuccini, 2014). Iso‐anisohydry is closely related to a plant's life‐history traits and survival strategy. Isohydry implies a more conservative water‐balance management strategy to prevent loss of water via transpiration (Sade, Gebremedhin, & Moshelion, 2012), which can protect plants from hydraulic failures and xylem embolism. Anisohydry maintains higher carbon assimilation at mild drought conditions and thus achieves an overall higher productivity (Urli, 2013). A previous study among species of Bornean rainforest found that anisohydry is more prevalent and reduces the risk of drought‐induced hydraulic failure (Kumagai & Porporato, 2012). However, satellite data suggested the contrary, that is, that isohydry is more dominant in wet areas (Li, 2017). These conflicting results reflect an important role that environment plays in shaping water relations and call for investigation of more aspects of plant hydraulic functions (Hochberg et al., 2018). More studies on specific systems and species are also needed to improve our understanding of species’ responses to drought in tropical forests under progressive climate change.

The heat shock applied in this study resulted in rapid closure of stomata in both well‐watered and drought‐stressed plants of both species, suggesting that drought and heat shock stress were additive in reducing *g_s_*. Transpiration via the stomatal aperture is a mechanism of heat regulation (Zhou, Chen, Li, & Chen, 2010). Previous studies have shown that many plants achieve heat loss via transpiration, with stomatal conductance either maintained at normal levels (Correia, 2018) or increased (Urban et al., 2017) to prevent leaf temperatures from reaching harmful levels. By contrast, our findings are similar to those reported in olive trees (Haworth, 2018), in which *g_s_* decreased with heat shock, and this could be a mechanism for plants adapted to low water availability to reduce the risk of xylem embolism. The level of heat stress tested here produced a conservative response in both *D. oliveri* and *D. cochinchinensis*. Stomatal responses may vary at different levels and durations of heat.

### 4.2 Leaf traits

We found no significant effect of drought or heat shock on leaf dry matter content (LDMC) or specific leaf area (SLA). LDMC and SLA are two leaf traits related to resource use and trade‐offs. High SLA or low LDMC implies rapid assimilation for growth and production, whereas low SLA or high LDMC implies efficient conservation of nutrients within structural and well‐protected tissues (Vitra, 2019). The temporal limitation in this study may not reflect effects in SLA as leaf development potentially operates over a longer timeframe. Previous studies mainly suggested that drought stress caused decreased SLA to reduce the surface area for transpiration and thus prevent water loss (Liu & Stützel, 2004; Pandey, Ramegowda, & Senthil‐Kumar, 2015).

We found a higher SLA in *D. oliveri* than *D. cochinchinensis*; however, *D. oliveri* was more isohydric in terms of hydraulic traits. This observation seems to contradict the above claim that SLA decreases plastically in response to drought. However, there is little understanding of variation of SLA across species. Poorter, Niinemets, Poorter, Wright, and Villar (2009) in their meta‐analysis suggested that SLA varies strongly with many factors including light, temperature, nutrient, and functional groups. An increase in SLA could be a compensating mechanism for reduced carbon allocation to the leaves during stress (Aspelmeier & Leuschner, 2006). In response to heat stress, increase in SLA could provide a mechanism for temperature regulation by increasing surface area (Pandey, Ramegowda, & Senthil‐Kumar, 2015). These features suggest that isohydric *D. oliveri* may suffer an impaired carbon assimilation under drought stress and compensate through associated leaf traits.

### 4.3 Photosynthetic pigments and carbon assimilation

We found that levels of chlorophyll a and carotenoids increased in *D. cochinchinensis* in response to drought and decreased in *D. oliveri*. Chlorophyll content is one of the most commonly used parameters to measure the severity of drought stress (Ying, 2015) as drought stress is thought to damage photosynthetic apparatus and diminish chlorophyll content (Fu & Huang, 2001). By maintaining lower chlorophyll content under severe drought conditions, plants are protected from photo‐oxidative damage by inhibiting photosynthesis and avoiding excess light excitation energy (Aranjuelo, Molero, Erice, Avice, & Nogué, 2011; Pintó‐Marijuan & Munné‐Bosch, 2014). As such, the decreasing trend of chlorophyll a in *D. oliveri* implies correspondence to drought avoidance as may be expected with an isohydric behavior. On the contrary, some studies have suggested that chlorophyll content is positively associated with drought tolerance and recovery (Chen, 2016) and that the potential photo‐oxidative damage can be remediated by increasing levels of carotenoids, which play a central role in the assembly of the light‐harvesting complex in the photosystem, regulate photomorphogenesis, and provide photoprotection (Munné‐Bosch & Alegre, 2000). Carotenoids are also precursors for two plant hormones, strigolactones, and abscisic acid (ABA) from carotenoid cleavage (Nisar, Li, Lu, Khin, & Pogson, 2015), while ABA has a dominant role in regulation of stomatal conductance in response to drought stress (Pirasteh‐Anosheh et al., 2016). Therefore, the contrasting trends of photosynthetic pigments further support the conclusion that *D. oliveri* avoids drought by reducing carbon assimilation and maintaining water potential, while *D. cochinchinensis* tolerates drought and maintains its productivity.

The higher levels of photosynthetic pigments associated with these increases in *D. cochinchinensis* may explain its higher total soluble sugars (TSS). Higher TSS may also provide an energy buffer to sustain metabolic activity during drought stress (Duan, 2015). The lower TSS in *D. oliveri* potentially implies a carbon limitation, which is commonly associated with isohydric species and could lead to carbon starvation in the long term (McDowell, 2008). Our finding that TSS increased in response to drought stress may suggest a mechanism of osmoregulation (Granda & Camarero, 2017), in which osmotically active soluble sugars can help maintain water potential and hydraulic integrity (O'Brien, Leuzinger, Philipson, Tay, & Hector, 2014).

In our study, we found little response specifically to heat shock in either species, thus potentially suggesting that these trees could have a higher heat threshold than 38°C for 4 hr. We did not observe any synergistic or antagonistic effect of both drought and heat stresses as in previous studies (Correia, 2018; Sheel et al., 2013; Zandalinas et al., 2018), except *g_s_*. There is an emerging interest in studying plant responses to a suite of stress factors, as different stresses may lead different signaling pathways to interact and conflict to produce novel physiological responses (Zandalinas et al., 2018).

### 4.4 Relating life‐history traits with potential ecological niches

*D. cochinchinensis* and *D. oliveri* are endemic to and geographically co‐occur in Cambodia, Laos, Thailand, and Vietnam. *D. cochinchinensis* is described as an intermediate pioneering species, characteristic of faster growth rate in young age (2000). Its anisohydric behavior, described here as maximizing carbon assimilation at the risk of hydraulic failure during drought, may be associated with its higher productivity for more efficient colonization in early ecological succession. This fits well with an earlier formulation by Smith and Huston (1989) that light‐demanding pioneers would optimize growth and outcompete shade‐tolerant species in drier habitats. Other studies also support the theory that pioneers run the risk of drought‐induced cavitation in order to maintain higher hydraulic efficiency and meet the water demand for photosynthesis and fast growth during drought (Markesteijn, Poorter, Bongers, Paz, & Sack, 2011; Markesteijn, Poorter, Paz, Sack, & Bongers, 2011). Such a trade‐off between short‐term growth and long‐term survival aligns with the spectrum from pioneers to shade‐tolerant species (Poorter & Bongers, 2006).

On the other hand, *D. oliveri* demonstrates a wider ecological amplitude within the deciduous forest, from relatively rich, deep soils to poor, shallow soils (Aerts et al., 2009). Aerts et al. (2009) suggested that *D. oliveri* can grow on shallow, eroded regosols where drought stress is persistent during the dry season. We report a more isohydric behavior in *D. oliveri* and hypothesize that it is more conservative in order to allow survival under low water availability of the region's deciduous forests.

The controlled nature of the experiment in this study allowed isolating the effects of multiple stressors and assessing their interactions (Seebacher & Franklin, 2012). Although the controlled experiment does not replicate the environmental and growth conditions in the field, its focus on seedlings shows close relevance to a crucial regeneration stage when mortality and selection pressures are high (Muscarella, 2013; Qie, 2019). We believe our results need further validation with field experiments and observations. At the same time, our results inform design of such field studies, for example by indicating the need to study multiple stressors and the divergent response strategies of individual species.

The contrasting hydraulic strategies of *D. cochinchinensis* and *D. oliveri* may reflect adaptation to their current ecological niche, but such dynamics may change drastically with changing climate and affect their survival and regeneration. The extent to which these water‐carbon trade‐offs affect growth and survival depends on species‐specific vulnerability to and capacity for recovery from hydraulic failure and carbon starvation. We recommend that research is needed to study responses and recovery to the extreme stresses at different life stages and under probable climate scenarios within their ranges.

## 5 CONCLUSION

To date, our understanding of physiology and ecological function in *D. cochinchinensis* and *D. oliveri* is poorly developed compared to that of their mating and genetic structure (Hartvig, 2018, 2020). This knowledge gap hinders informed decision‐making for the conservation and restoration of these valuable species. We present the first study on hydraulic traits and carbon assimilation of *D. cochinchinensis* and *D. oliveri* in their seedling stages and characterize their iso‐anisohydric behavior in response to drought and heat stresses. We suggest potential thresholds and interactions of drought and heat stresses, which will open the opportunity for further studies to gain a better understanding of their physiology and stress response. Such an approach would also be beneficial to studying stress responses in other *Dalbergia* species to similarly inform their conservation and use.

*Dalbergia* species regenerate naturally when circumstances permit, but are also suited to use for planting in restoring degraded forests and restoring deforested sites (Koonkhunthod, Sakurai, & Tanaka, 2007). Seedling establishment is a crucial life stage of trees and an understanding of their stress responses will contribute to appropriate site selection and effective long‐term regeneration (Abiyu, Teketay, Glatzel, & Gratzer, 2016; Fontaine, 2007), especially with regard to restoration efforts and climate change.

## CONFLICT OF INTERESTS

The authors declare no competing interests.

## AUTHOR CONTRIBUTION

**Tin Hang Hung:** Conceptualization (equal); Funding acquisition (equal); Investigation (equal); Methodology (equal); Supervision (equal); Writing‐original draft (equal); Writing‐review & editing (equal). **Rosemary Gooda:** Data curation (equal); Formal analysis (equal); Investigation (equal); Visualization (equal); Writing‐original draft (equal); Writing‐review & editing (equal). **Gabriele Rizzuto:** Methodology (equal); Validation (equal); Writing‐review & editing (equal). **Thea So:** Funding acquisition (equal); Project administration (equal); Resources (equal); Validation (equal); Writing‐review & editing (equal). **Bansa Thammavong:** Funding acquisition (equal); Project administration (equal); Resources (equal); Validation (equal); Writing‐review & editing (equal). **Hoa Thi Tran:** Funding acquisition (equal); Project administration (equal); Resources (equal); Validation (equal); Writing‐review & editing (equal). **Riina Jalonen:** Funding acquisition (equal); Project administration (equal); Validation (equal); Writing‐review & editing (equal). **David H. Boshier:** Funding acquisition (equal); Project administration (equal); Supervision (equal); Validation (equal); Writing‐review & editing (equal). **John J. MacKay:** Conceptualization (equal); Funding acquisition (equal); Project administration (equal); Supervision (equal); Writing‐review & editing (equal).

## Supporting information

Table S1‐S11

## Data Availability

The research materials supporting this publication can be publicly accessed in Dryad (https://doi.org/10.5061/dryad.v6wwpzgt1).

## References

[ece36744-bib-0001] Abiyu, A. , Teketay, D. , Glatzel, G. , & Gratzer, G. (2016). Seed production, seed dispersal and seedling establishment of two afromontane tree species in and around a church forest: Implications for forest restoration. Forest Ecosystems, 3, 1–10.

[ece36744-bib-0002] Aerts, R. , Volkaert, H. , Roongruangsree, N. , Roongruangsree, U. T. , Swennen, R. , & Muys, B. (2009). Site requirements of the endangered rosewood *Dalbergia oliveri* in a tropical deciduous forest in northern Thailand. Forest Ecology and Management, 259, 117–123.

[ece36744-bib-0003] Aitken, S. N. , Yeaman, S. , Holliday, J. A. , Wang, T. , & Curtis‐McLane, S. (2008). Adaptation, migration or extirpation: Climate change outcomes for tree populations. Evolutionary Applications, 1, 95–111. 25567494 10.1111/j.1752-4571.2007.00013.x PMC3352395

[ece36744-bib-0004] Allen, C. D. , Breshears, D. D. , & McDowell, N. G. (2015). On underestimation of global vulnerability to tree mortality and forest die‐off from hotter drought in the Anthropocene. Ecosphere, 6, 1–55. 10.1890/ES15-00203.1

[ece36744-bib-0005] Allen, C. D. , Macalady, A. K. , Chenchouni, H. , Bachelet, D. , McDowell, N. , Vennetier, M. , … Cobbt, N. (2010). A global overview of drought and heat‐induced tree mortality reveals emerging climate change risks for forests. Forest Ecology and Management, 259, 660–684.

[ece36744-bib-0006] APFORGEN (2018). Conserving Rosewood genetic resources for resilient livelihoods in the Mekong: Project inception workshop report. .

[ece36744-bib-0007] Aranjuelo, I. , Molero, G. , Erice, G. , Avice, J. C. , & Nogué, S. (2011). Plant physiology and proteomics reveals the leaf response to drought in alfalfa (*Medicago sativa* L.). Journal of Experimental Botany, 62, 111–123. 20797998 10.1093/jxb/erq249 PMC2993905

[ece36744-bib-0008] Aspelmeier, S. , & Leuschner, C. (2006). Genotypic variation in drought response of silver birch (Betula pendula Roth): Leaf and root morphology and carbon partitioning. Trees – Structure and Function, 20, 42–52.

[ece36744-bib-0009] Bates, D. M. , & Watts, D. G. (1988). Nonlinear Regression analysis and its applications. Hoboken, NJ: Wiley.

[ece36744-bib-0010] Bergel‐Landefeldt, U. (1936). Das Wasserhaushalt der Alpenpflanzen (Bibliotheca Botanica Vol. 115). Schweizerbartsche Verlagsbuchhandlung.

[ece36744-bib-0011] Bhagwat, R. M. , Dholakia, B. B. , Kadoo, N. Y. , Balasundaran, M. , & Gupta, V. S. (2015). Two new potential barcodes to discriminate *Dalbergia* species. PLoS One, 10, 1–18. 10.1371/journal.pone.0142965 PMC4646644 26569490

[ece36744-bib-0012] Brodribb, T. J. , Powers, J. , Cochard, H. , & Choat, B. (2020). Hanging by a thread? Forests and drought. Science, 368, 261–266. 10.1126/science.aat7631 32299945

[ece36744-bib-0013] Canham, C. D. , & Murphy, L. (2016). The demography of tree species response to climate: Seedling recruitment and survival. Ecosphere, 7, e01424 10.1002/ecs2.1424

[ece36744-bib-0014] CDRI (2014). Community forestry for sustainable forest management and livelihoods: A case study of Osoam Community FOrest users group. Cambodia Developmental Review, 18 1–9.

[ece36744-bib-0015] Chaves, M. M. , Pereira, J. S. , Maroco, J. , Rodrigues, M. L. , Ricardo, C. P. P. , Osório, M. L. , … Pinheiro, C. (2002). How plants cope with water stress in the field. Photosynthesis and growth. Annals of Botany, 89, 907–916. 12102516 10.1093/aob/mcf105 PMC4233809

[ece36744-bib-0016] Chen, D. , Wang, S. , Cao, B. , Cao, D. , Leng, G. , Li, H. , … Deng, X. (2016). Genotypic variation in growth and physiological response to drought stress and re‐watering reveals the critical role of recovery in drought adaptation in maize seedlings. Frontiers in Plant Science, 6, 1241. 26793218 10.3389/fpls.2015.01241 PMC4709455

[ece36744-bib-0017] Correia, B. , Hancock, R. D. , Amaral, J. , Gomez‐Cadenas, A. , Valledor, L. , & Pinto, G. (2018). Combined drought and heat activates protective responses in *Eucalyptus globulus* that are not activated when subjected to drought or heat stress alone. Frontiers in Plant Science, 9, 819. 29973941 10.3389/fpls.2018.00819 PMC6019450

[ece36744-bib-0018] de Terra, M. C. N. S. , dos Santos, R. M. , Prado Júnior, J. A. , de Mello, J. M. , Soares Scolforo, J. R. , Leite Fontes, M. A. , & ter Steege, H. (2018). Water availability drives gradients of tree diversity, structure and functional traits in the Atlantic–Cerrado–Caatinga transition. Brazilian Journal of Plant Ecology, 11, 803–814.

[ece36744-bib-0019] Duan, H. , O'Grady, A. P. , Duursma, R. A. , Choat, B. , Huang, G. , Smith, R. A. , … Tissue, D. T. (2015). Drought responses of two gymnosperm species with contrasting stomatal regulation strategies under elevated [CO2] and temperature. Tree Physiology, 35, 756–770. 26063706 10.1093/treephys/tpv047

[ece36744-bib-0020] EIA (2017). The Rosewood Racket: China’s billion dollar illegal timber trade and the devastation of Nigeria’s forests.Environmental Investigation Agency.

[ece36744-bib-0021] Estravis‐Barcala, M. , Mattera, M. G. , Soliani, C. , Bellora, N. , Opgenoorth, L. , Heer, K. , & Arana, M. V. (2019). Molecular bases of responses to abiotic stress in trees. Journal of Experimental Botany, 71(13), 3765–3779. 10.1093/jxb/erz532 PMC7316969 31768543

[ece36744-bib-0022] FAO (2017). Climate‐smart crop production practices and technologies In Climate smart agriculture sourcebook.Food and Agriculture Organization of the United Nations http://www.fao.org/climate‐smart‐agriculture‐sourcebook/production‐resources/module‐b1‐crops/chapter‐b1‐2/en/

[ece36744-bib-0023] Fontaine, M. , Aerts, R. , Özkan, K. , Mert, A. , Gülsoy, S. , Süel, H. , … Muys, B. (2007). Elevation and exposition rather than soil types determine communities and site suitability in Mediterranean mountain forests of southern Anatolia, Turkey. Forest Ecology and Management, 247, 18–25.

[ece36744-bib-0024] Fu, J. , & Huang, B. (2001). Involvement of antioxidants and lipid peroxidation in the adaptation of two cool‐season grasses to localized drought stress. Environmental and Experimental Botany, 45, 105–114. 11275219 10.1016/s0098-8472(00)00084-8

[ece36744-bib-0025] Granda, E. , & Camarero, J. J. (2017). Drought reduces growth and stimulates sugar accumulation: New evidence of environmentally driven non‐structural carbohydrate use. Tree Physiology, 37, 997–1000. 28903526 10.1093/treephys/tpx097

[ece36744-bib-0026] Hartvig, I. , So, T. , Changtragoon, S. , Tran, H. T. , Bouamanivong, S. , Ogden, R. , … Kjær, E. D. (2020). Conservation genetics of the critically endangered Siamese rosewood (*Dalbergia cochinchinensis*): Recommendations for management and sustainable use. Conservation Genetics, 21(4), 677–692. 10.1007/s10592-020-01279-1

[ece36744-bib-0027] Hartvig, I. , So, T. , Changtragoon, S. , Tran, H. T. , Bouamanivong, S. , Theilade, I. , … Nielsen, L. R. (2018). Population genetic structure of the endemic rosewoods *Dalbergia cochinchinensis* and *D. oliveri* at a regional scale reflects the Indochinese landscape and life‐history traits. Ecology and Evolution, 8, 530–545. 29321891 10.1002/ece3.3626 PMC5756888

[ece36744-bib-0028] Haworth, M. , Marino, G. , Brunetti, C. , Killi, D. , De Carlo, A. , & Centritto, M. (2018). The impact of heat stress and water deficit on the photosynthetic and stomatal physiology of olive (*Olea europaea* L.)—A case study of the 2017 heat wave. Plants, 7, 76 10.3390/plants7040076 PMC6313851 30241389

[ece36744-bib-0029] Hochberg, U. , Rockwell, F. E. , Holbrook, N. M. , & Cochard, H. (2018). Iso/Anisohydry: A plant‐environment interaction rather than a simple hydraulic trait. Trends in Plant Science, 23, 112–120. 29223922 10.1016/j.tplants.2017.11.002

[ece36744-bib-0030] Hughes, A. C. (2017). Understanding the drivers of Southeast Asian biodiversity loss. Ecosphere, 8, e01624 10.1002/ecs2.1624

[ece36744-bib-0031] Jalonen, R. , Valette, M. , Boshier, D. , Duminil, J. , & Thomas, E. (2018). Forest and landscape restoration severely constrained by a lack of attention to the quantity and quality of tree seed: Insights from a global survey. Conservation Letters, 11, e12424.

[ece36744-bib-0032] Kaewkrom, P. , Gajaseni, J. , Jordan, C. F. , & Gajaseni, N. (2005). Floristic regeneration in five types of teak plantations in Thailand. Forest Ecology and Management, 210, 351–361.

[ece36744-bib-0033] Klein, T. (2014). The variability of stomatal sensitivity to leaf water potential across tree species indicates a continuum between isohydric and anisohydric behaviours. Functional Ecology, 28, 1313–1320. 10.1111/1365-2435.12289

[ece36744-bib-0034] Koonkhunthod, N. , Sakurai, K. , & Tanaka, S. (2007). Composition and diversity of woody regeneration in a 37‐year‐old teak (*Tectona grandis* L.) plantation in Northern Thailand. Forest Ecology and Management, 247, 246–254.

[ece36744-bib-0035] Kumagai, T. , & Porporato, A. (2012). Strategies of a Bornean tropical rainforest water use as a function of rainfall regime: Isohydric or anisohydric? Plant, Cell and Environment, 35, 61–71. 10.1111/j.1365-3040.2011.02428.x 21933196

[ece36744-bib-0036] Lavoie‐Lamoureux, A. , Sacco, D. , Risse, P.‐A. , & Lovisolo, C. (2017). Factors influencing stomatal conductance in response to water availability in grapevine: A meta‐analysis. Physiologia Plantarum, 159, 468–482. 27859326 10.1111/ppl.12530

[ece36744-bib-0037] Li, X. , Yang, Y. , Sun, X. , Lin, H. , Chen, J. , Ren, J. , … Yang, Y. (2014). Comparative physiological and proteomic analyses of poplar (*Populus yunnanensis*) plantlets exposed to high temperature and drought. PLoS One, 9, e107605 10.1371/journal.pone.0107605 25225913 PMC4167240

[ece36744-bib-0038] Li, Y. , Guan, K. , Gentine, P. , Konings, A. G. , Meinzer, F. C. , Kimball, J. S. , … Good, S. P. (2017). Estimating Global Ecosystem Isohydry/Anisohydry using active and passive microwave satellite data. Journal of Geophysical Research: Biogeosciences, 122, 3306–3321.

[ece36744-bib-0039] Li, Y. , Zhao, M. , Motesharrei, S. , Mu, Q. , Kalnay, E. , & Li, S. (2015). Local cooling and warming effects of forests based on satellite observations. Nature Communications, 6, 1–8. 10.1038/ncomms7603 PMC4389237 25824529

[ece36744-bib-0040] Liu, F. , & Stützel, H. (2004). Biomass partitioning, specific leaf area, and water use efficiency of vegetable amaranth (*Amaranthus* spp.) in response to drought stress. Scientia Horticulturae, 102, 15–27.

[ece36744-bib-0041] Luoma‐aho, T. , Hong, L. T. , Ramanatha Rao, V. , & Sim, H. C. (2003). Forest genetic resources conservation and management: Proceedings of the Asia Pacific Forest Genetic Resources Programme (APFORGEN) Inception Workshop.

[ece36744-bib-0042] Maningo, E. V. , & Thea, S. Regional project for promotion of forest rehabilitation in Cambodia and Vietnam through demonstration models and improvement of seed supply system: Lesson learned.

[ece36744-bib-0043] Markesteijn, L. , Poorter, L. , Bongers, F. , Paz, H. , & Sack, L. (2011). Hydraulics and life history of tropical dry forest tree species: Coordination of species’ drought and shade tolerance. New Phytologist, 191, 480–495. 21477008 10.1111/j.1469-8137.2011.03708.x

[ece36744-bib-0044] Markesteijn, L. , Poorter, L. , Paz, H. , Sack, L. , & Bongers, F. (2011). Ecological differentiation in xylem cavitation resistance is associated with stem and leaf structural traits. Plant, Cell and Environment, 34, 137–148. 10.1111/j.1365-3040.2010.02231.x 20946587

[ece36744-bib-0045] Martínez‐Vilalta, J. , Poyatos, R. , Aguadé, D. , Retana, J. , & Mencuccini, M. (2014). A new look at water transport regulation in plants. New Phytologist, 204, 105–115. 24985503 10.1111/nph.12912

[ece36744-bib-0046] McDowell, N. , Allen, C. D. , Anderson‐Teixeira, K. , Brando, P. , Brienen, R. , Chambers, J. , … Xu, X. (2018). Drivers and mechanisms of tree mortality in moist tropical forests. New Phytologist, 219, 851–869. 10.1111/nph.15027 29451313

[ece36744-bib-0047] McDowell, N. , Pockman, W. T. , Allen, C. D. , Breshears, D. D. , Cobb, N. , Kolb, T. , … Yepez, E. A. (2008). Mechanisms of plant survival and mortality during drought: Why do some plants survive while others succumb to drought? New Phytologist, 178, 719–739. 18422905 10.1111/j.1469-8137.2008.02436.x

[ece36744-bib-0048] MRC (2010). State of the basin report 2010.

[ece36744-bib-0049] Munné‐Bosch, S. , & Alegre, L. (2000). Changes in carotenoids, tocopherols and diterpenes during drought and recovery, and the biological significance of chlorophyll loss in *Rosmarinus officinalis* plants. Planta, 210, 925–931. 10.1007/s004250050699 10872224

[ece36744-bib-0050] Muscarella, R. , Uriarte, M. , Forero Montaña, J. , Comita, L. S. , Swenson, N. G. , Thompson, J. , & Zimmerman, J. K. (2013). Life‐history trade‐offs during the seed‐to‐seedling transition in a subtropical wet forest community. Journal of Ecology, 101, 171–182.

[ece36744-bib-0051] Nisar, N. , Li, L. , Lu, S. , Khin, N. C. , & Pogson, B. J. (2015). Carotenoid metabolism in plants. Molecular Plant, 8, 68–82. 10.1016/j.molp.2014.12.007 25578273

[ece36744-bib-0052] O'Brien, M. J. , Leuzinger, S. , Philipson, C. D. , Tay, J. , & Hector, A. (2014). Drought survival of tropical tree seedlings enhanced by non‐structural carbohydrate levels. Nature Climate Change, 4, 710–714.

[ece36744-bib-0053] Osaki, M. , Shinano, T. , & Tadano, T. (1991). Redistribution of carbon and nitrogen compounds from the shoot to the harvesting organs during maturation in field crops. Soil Science and Plant Nutrition, 37, 117–128.

[ece36744-bib-0054] Pandey, P. , Ramegowda, V. , & Senthil‐Kumar, M. (2015). Shared and unique responses of plants to multiple individual stresses and stress combinations: Physiological and molecular mechanisms. Frontiers in Plant Science, 6, 723 10.3389/fpls.2015.00723 26442037 PMC4584981

[ece36744-bib-0055] Pérez‐Harguindeguy, N. , Díaz, S. , Garnier, E. , Lavorel, S. , Poorter, H. , Jaureguiberry, P. , … Cornelissen, J. H. C. (2013). New handbook for standardised measurement of plant functional traits worldwide. Australian Journal of Botany, 61, 167–234.

[ece36744-bib-0056] Pintó‐Marijuan, M. , & Munné‐Bosch, S. (2014). Photo‐oxidative stress markers as a measure of abiotic stress‐induced leaf senescence: Advantages and limitations. Journal of Experimental Botany, 65, 3845–3857. 24683180 10.1093/jxb/eru086

[ece36744-bib-0057] Pirasteh‐Anosheh, H. , Saed‐Moucheshi, A. , Pakniyat, H. , & Pessarakli, M. (2016). Stomatal responses to drought stress Ahmad P. (Ed), In Water stress and crop plants: A sustainable approach (vols. 1–2, pp. 24–40). Hoboken, NJ: Wiley.

[ece36744-bib-0058] Pollastrini, M. , Puletti, N. , Selvi, F. , Iacopetti, G. , & Bussotti, F. (2019). Widespread crown defoliation after a drought and heat wave in the forests of Tuscany (Central Italy) and Their Recovery—A Case Study From Summer 2017. Frontiers in Forests and Global Change, 2, 74.

[ece36744-bib-0059] Polle, A. , Chen, S. L. , Eckert, C. , & Harfouche, A. (2019). Engineering drought resistance in forest trees. Frontiers in Plant Science, 9, 1875 10.3389/fpls.2018.01875 30671067 PMC6331418

[ece36744-bib-0060] Poorter, H. , Niinemets, Ü. , Poorter, L. , Wright, I. J. , & Villar, R. (2009). Causes and consequences of variation in leaf mass per area (LMA): A meta‐analysis. New Phytologist, 182, 565–588. 19434804 10.1111/j.1469-8137.2009.02830.x

[ece36744-bib-0061] Poorter, L. , & Bongers, F. (2006). Leaf Traits are good predictors of plant performance across 53 Rain Forest Species. Ecology, 87, 1733–1743. 10.1890/0012-9658(2006)87[1733:LTAGPO]2.0.CO;2 16922323

[ece36744-bib-0062] Qie, L. , Telford, E. M. , Massam, M. R. , Tangki, H. , Nilus, R. , Hector, A. , & Ewers, R. M. (2019). Drought cuts back regeneration in logged tropical forests. Environmental Research Letters, 14(4), 45012 10.1088/1748-9326/ab0783

[ece36744-bib-0063] Rizhsky, L. , Liang, H. , & Mittler, R. (2002). The combined effect of drought stress and heat shock on gene expression in Tobacco. Plant Physiology, 130, 1143–1151. 12427981 10.1104/pp.006858 PMC166635

[ece36744-bib-0064] Sade, N. , Gebremedhin, A. , & Moshelion, M. (2012). Risk‐taking plants: Anisohydric behavior as a stress‐resistance trait. Plant Signaling & Behavior, 7, 767–770. 10.4161/psb.20505 22751307 PMC3583960

[ece36744-bib-0065] Sakai, A. , Visaratana, T. , Vacharangkura, T. , Thai‐ngam, R. , Tanaka, N. , Ishizuka, M. , & Nakamura, S. (2009). Effect of species and spacing of fast‐growing nurse trees on growth of an indigenous tree, Hopea odorata Roxb., in northeast Thailand. Forest Ecology and Management, 257, 644–652.

[ece36744-bib-0066] Schneider, C. A. , Rasband, W. S. , & Eliceiri, K. W. (2012). NIH Image to ImageJ: 25 years of image analysis. Nature Methods, 9, 671–675. 10.1038/nmeth.2089 22930834 PMC5554542

[ece36744-bib-0067] Seebacher, F. , & Franklin, C. E. (2012). Determining environmental causes of biological effects: The need for a mechanistic physiological dimension in conservation biology. Philosophical Transactions of the Royal Society B: Biological Sciences, 367, 1607–1614. 10.1098/rstb.2012.0036 PMC3350663 22566670

[ece36744-bib-0068] Sheel, B. , Göran, H. , Löfvenius, M. O. , & Marie‐Charlotte, N. (2013). Additive and antagonistic impacts of drought and herbivory on *Pinus Sylvestris*: Leaf, tissue and whole‐plant responses and recovery. Tree Physiology, 33, 451–463. 23525156 10.1093/treephys/tpt019

[ece36744-bib-0069] Sims, D. A. , & Gamon, J. A. (2002). Relationships between leaf pigment content and spectral reflectance across a wide range of species, leaf structures and developmental stages. Remote Sensing of Environment, 81, 337–354.

[ece36744-bib-0070] Sitch, S. et al (2008). Evaluation of the terrestrial carbon cycle, future plant geography and climate‐carbon cycle feedbacks using five Dynamic Global Vegetation Models (DGVMs). Global Change Biology, 14, 2015–2039.

[ece36744-bib-0071] Smith, T. , & Huston, M. (1989). A theory of the spatial and temporal dynamics of plant communities. Vegetatio, 83, 49–69. 10.1007/BF00031680

[ece36744-bib-0072] So, N. V. (2000).The potential of local tree species to accelerate natural forest succession on marginal grasslands in Southern Vietnam In Elliot S., Kerby J., & Blakesly K. et al (Eds.), Proceedings of the Workshop on Forest Restoration for Wildlife Restoration for Wildlife Conservation. International Tropical Timber Organization and The Forest Restoration Unit, Chiang Mai University.

[ece36744-bib-0073] Stéfanon, M. , Drobinski, P. , D’Andrea, F. , Lebeaupin‐Brossier, C. , & Bastin, S. (2014). Soil moisture‐temperature feedbacks at meso‐scale during summer heat waves over Western Europe. Climate Dynamics, 42, 1309–1324.

[ece36744-bib-0074] Tanaka, N. , Kume, T. , Yoshifuji, N. , Tanaka, K. , Takizawa, H. , Shiraki, K. , … Suzuki, M. (2008). A review of evapotranspiration estimates from tropical forests in Thailand and adjacent regions. Agricultural & Forest Meteorology, 148, 807–819.

[ece36744-bib-0075] Teskey, R. , Wertin, T. , Bauweraerts, I. , Ameye, M. , McGuire, M. A. , … Steppe, K. (2015). Responses of tree species to heat waves and extreme heat events. Plant, Cell and Environment, 38, 1699–1712. 10.1111/pce.12417 25065257

[ece36744-bib-0076] Urban, J. , Ingwers, M. , McGuire, M. A. , & Teskey, R. O. (2017). Stomatal conductance increases with rising temperature. Plant Signaling & Behavior, 12, e1356534 10.1080/15592324.2017.1356534 28786730 PMC5616154

[ece36744-bib-0077] Urli, M. , Porté, A. J. , Cochard, H. , Guengant, Y. , Burlett, R. , & Delzon, S. (2013). Xylem embolism threshold for catastrophic hydraulic failure in angiosperm trees. Tree Physiology, 33, 672–683. 23658197 10.1093/treephys/tpt030

[ece36744-bib-0078] Vatanparast, M. , Klitgård, B. B. , Adema, F. A. C. B. , Pennington, R. T. , Yahara, T. , & Kajita, T. (2013). First molecular phylogeny of the pantropical genus Dalbergia: Implications for infrageneric circumscription and biogeography. South African Journal of Botany, 89, 143–149.

[ece36744-bib-0079] Vitra, A. , Deléglise, C. , Meisser, M. , Risch, A. C. , Signarbieux, C. , Lamacque, L. , … Mariotte, P. (2019). Responses of plant leaf economic and hydraulic traits mediate the effects of early‐ and late‐season drought on grassland productivity. AoB Plants, 11, 23 10.1093/aobpla/plz023 PMC6499892 31065332

[ece36744-bib-0080] Winfield, K. , Scott, M. , & Graysn, C. (2016). Global status of *Dalbergia* and *Pterocarpus* rosewood producing species in trade. In *Convention on International Trade in Endangered Species 17th Conference of Parties – Johannesburg*.

[ece36744-bib-0081] Ying, Y. Q. , Song, L. L. , Jacobs, D. F. , Mei, L. , Liu, P. , Jin, S. H. , & Wu, J. S. (2015). Physiological response to drought stress in *Camptotheca acuminata* seedlings from two provenances. Frontiers in Plant Science, 6, 1–8. 26052334 10.3389/fpls.2015.00361 PMC4440367

[ece36744-bib-0082] Zandalinas, S. I. , Mittler, R. , Balfagón, D. , Arbona, V. , & Gómez‐Cadenas, A. (2018). Plant adaptations to the combination of drought and high temperatures. Physiologia Plantarum, 162, 2–12. 10.1111/ppl.12540 28042678

[ece36744-bib-0083] Zhou, H. H. , Chen, Y. N. , Li, W. H. , & Chen, Y. P. (2010). Photosynthesis of *Populus euphratica* in relation to groundwater depths and high temperature in arid environment, northwest China. Photosynthetica, 48, 257–268. 10.1007/s11099-010-0032-5

