# Supplementary material for: Physiological responses of rosewoods Dalbergia cochinchinensis and D. oliveri under drought and heat stresses
Source: Ecol Evol. 2020 Sep 9;10(19):10872–85. doi: 10.1002/ece3.6744 (PMC7548189; doi:10.1002/ece3.6744)
Supplement: Supplementary file 1 — Table S1‐S11 [file ECE3-10-10872-s001.docx]

Supplementary Table 1

| **Factor** | **Df** | **Sum sq** | **Mean sq** | **F value** | **Pr(> F)** |
| --- | --- | --- | --- | --- | --- |
| Drought | 1 | 1408.0 | 1407.95 | 8.0014 | 0.0134* |
| Error (main-plot) | 14 | 2463.5 | 175.96 |  |  |
| Block | 15 | 5119.6 | 341.31 | 9.8187 | 0.0001507* |
| Species | 1 | 560.9 | 560.94 | 16.1371 | 0.0017083* |
| Species : drought | 1 | 9.1 | 9.09 | 0.2615 | 0.6183585 |
| Error | 12 | 417.1 | 34.76 |  |  |

Supplementary Table 2

| **Factor** | **Df** | **Sum sq** | **Mean sq** | **F value** | **Pr(> F)** |
| --- | --- | --- | --- | --- | --- |
| Drought | 1 | 12009 | 12008.7 | 1.2234 | 0.2873 |
| Error (main-plot) | 14 | 137426 | 9816.1 |  |  |
| Block | 15 | 188827 | 12588 | 7.1974 | 0.0007127* |
| Species | 1 | 151109 | 151109 | 86.3965 | 7.844e-07* |
| Species : drought | 1 | 186 | 186 | 0.1062 | 0.7500877 |
| Error (split-plot) | 12 | 20988 | 1749 |  |  |

Supplementary Table 3

| **Factor** | **Df** | **Sum sq** | **Mean sq** | **F value** | **Pr(> F)** |
| --- | --- | --- | --- | --- | --- |
| Drought | 1 | 1.39741 | 1.39741 | 38.171 | 2.401e-05* |
| Error (main-plot) | 14 | 0.51253 | 0.03661 |  |  |
| Heat shock | 1 | 17.0342 | 17.0342 | 1112.0253 | 2.936e-15* |
| Block | 15 | 12.0692 | 0.8046 | 5.2915 | 1.576e-06* |
| Species | 1 | 4.0044 | 4.0044 | 26.3351 | 3.366e-06* |
| Day | 2 | 1.4667 | 0.7334 | 4.8229 | 0.011482* |
| Drought : heat shock | 1 | 1.9638 | 1.9638 | 12.9148 | 0.000666* |
| Day : drought | 2 | 2.0458 | 1.0229 | 6.7270 | 0.002335* |
| Day : heat shock | 2 | 0.4135 | 0.2067 | 1.3597 | 0.264674 |
| Species : drought | 1 | 0.0034 | 0.0034 | 0.0223 | 0.881672 |
| Species : heat shock | 1 | 0.2863 | 0.2863 | 1.8827 | 0.175225 |
| Species : day | 2 | 0.1423 | 0.0712 | 0.4681 | 0.628517 |
| Day : drought : heat shock | 2 | 0.5062 | 0.2531 | 1.6646 | 0.198029 |
| Species : drought : heat shock | 1 | 0.5349 | 0.5349 | 3.5176 | 0.065669 |
| Species : day : drought | 2 | 0.3421 | 0.1711 | 1.1250 | 0.331503 |
| Speces : day : heat shock | 2 | 0.3117 | 0.1558 | 1.0248 | 0.365172 |
| Species : day : drought : heat shock | 2 | 0.2149 | 0.1075 | 0.7067 | 0.497410 |
| Error (split-plot) | 59 | 8.9713 | 0.1521 |  |  |

Supplementary Table 4

| **Factor** | **Df** | **Sum sq** | **Mean sq** | **F value** | **Pr(> F)** |
| --- | --- | --- | --- | --- | --- |
| Drought | 1 | 8.7392 | 8.7392 | 5.1517 | 0.03955* |
| Error (main-plot) | 14 | 23.7492 | 1.6964 |  |  |
| Heat shock | 1 | 7.34 | 7.341 | 0.7939 | 0.376529 |
| Block | 15 | 187.21 | 12.480 | 1.3497 | 0.203269 |
| Species | 1 | 13.46 | 13.465 | 1.4562 | 0.232354 |
| Day | 2 | 260.73 | 130.364 | 14.0987 | 9.892e-06* |
| Drought : heat shock | 1 | 7.34 | 7.341 | 0.7939 | 0.376529 |
| Day : drought | 2 | 202.06 | 101.029 | 10.9261 | 9.188e-05* |
| Day : heat shock | 2 | 18.42 | 9.209 | 0.9960 | 0.375480 |
| Species : drought | 1 | 82.32 | 82.320 | 8.9028 | 0.004137* |
| Species : heat shock | 1 | 16.42 | 16.424 | 1.7762 | 0.187738 |
| Species : day | 2 | 10.55 | 5.274 | 0.5704 | 0.568387 |
| Day : drought : heat shock | 2 | 20.77 | 10.387 | 1.1234 | 0.332035 |
| Species : drought : heat shock | 1 | 30.75 | 30.749 | 3.3255 | 0.073281 |
| Species : day : drought | 2 | 45.14 | 22.569 | 2.4408 | 0.095842 |
| Speces : day : heat shock | 2 | 3.74 | 1.870 | 0.2022 | 0.817486 |
| Species : day : drought : heat shock | 2 | 0.33 | 0.167 | 0.0181 | 0.982075 |
| Error (split-plot) | 59 | 545.55 | 9.247 |  |  |

Supplementary Table 5

| **Factor** | **Df** | **Sum sq** | **Mean sq** | **F value** | **Pr(> F)** |
| --- | --- | --- | --- | --- | --- |
| Drought | 1 | 0.0002297 | 0.00022972 | 0.4279 | 0.5236 |
| Error (main-plot) | 14 | 0.0075165 | 0.00053689 |  |  |
| Heat shock | 1 | 0.005667 | 0.0056668 | 1.8272 | 0.18162 |
| Block | 15 | 0.046698 | 0.0031132 | 1.0038 | 0.46386 |
| Species | 1 | 0.001281 | 0.0012807 | 0.4130 | 0.52296 |
| Day | 2 | 0.016766 | 0.0083830 | 2.7030 | 0.07530 |
| Drought : heat shock | 1 | 0.003203 | 0.0032033 | 1.0329 | 0.31363 |
| Day : drought | 2 | 0.012903 | 0.0064513 | 2.0802 | 0.13398 |
| Day : heat shock | 2 | 0.037019 | 0.0185094 | 5.9682 | 0.00436* |
| Species : drought | 1 | 0.000067 | 0.0000667 | 0.0215 | 0.88395 |
| Species : heat shock | 1 | 0.002547 | 0.0025474 | 0.8214 | 0.36846 |
| Species : day | 2 | 0.003652 | 0.0018262 | 0.5888 | 0.55820 |
| Day : drought : heat shock | 2 | 0.006750 | 0.0033749 | 1.0882 | 0.32349 |
| Species : drought : heat shock | 1 | 0.000085 | 0.0000847 | 0.0273 | 0.86931 |
| Species : day : drought | 2 | 0.001544 | 0.0007722 | 0.2490 | 0.78040 |
| Speces : day : heat shock | 2 | 0.008646 | 0.0043229 | 1.3939 | 0.25616 |
| Species : day : drought : heat shock | 2 | 0.006402 | 0.0032012 | 1.0322 | 0.36257 |
| Error (split-plot) | 59 | 0.182980 | 0.0031014 |  |  |

Supplementary Table 6

| **Factor** | **Df** | **Sum sq** | **Mean sq** | **F value** | **Pr(> F)** |
| --- | --- | --- | --- | --- | --- |
| Drought | 1 | 0.004837 | 0.0048374 | 0.3514 | 0.5628 |
| Error (main-plot) | 14 | 0.192709 | 0.0137649 |  |  |
| Heat shock | 1 | 0.0273 | 0.0273 | 0.3786 | 0.5407 |
| Block | 15 | 1.3237 | 0.0882 | 1.2230 | 0.2812 |
| Species | 1 | 5.8698 | 5.8698 | 81.3485 | 1.061e-12* |
| Day | 2 | 0.2332 | 0.1166 | 1.6157 | 0.2074 |
| Drought : heat shock | 1 | 0.1062 | 0.1062 | 1.4719 | 0.2299 |
| Day : drought | 2 | 0.5980 | 0.2990 | 4.1439 | 0.0207* |
| Day : heat shock | 2 | 0.2757 | 0.1379 | 1.9107 | 0.1570 |
| Species : drought | 1 | 0.0020 | 0.0020 | 0.0277 | 0.8683 |
| Species : heat shock | 1 | 0.0048 | 0.0048 | 0.0669 | 0.7969 |
| Species : day | 2 | 0.0013 | 0.0007 | 0.0090 | 0.9910 |
| Day : drought : heat shock | 2 | 0.1124 | 0.0562 | 0.7790 | 0.4635 |
| Species : drought : heat shock | 1 | 0.0078 | 0.0078 | 0.1087 | 0.7428 |
| Species : day : drought | 2 | 0.0344 | 0.0172 | 0.2386 | 0.7885 |
| Speces : day : heat shock | 2 | 0.1742 | 0.0871 | 1.2068 | 0.3064 |
| Species : day : drought : heat shock | 2 | 0.2163 | 0.1081 | 1.4987 | 0.2318 |
| Error (split-plot) | 59 | 4.2572 | 0.0722 |  |  |

Supplementary Table 7

| **Factor** | **Df** | **Sum sq** | **Mean sq** | **F value** | **Pr(> F)** |
| --- | --- | --- | --- | --- | --- |
| Drought | 1 | 0.00618 | 0.0061774 | 0.2272 | 0.641 |
| Error (main-plot) | 14 | 0.38069 | 0.0271921 |  |  |
| Heat shock | 1 | 0.1606 | 0.16062 | 0.4258 | 0.51659 |
| Block | 15 | 2.4567 | 0.16378 | 0.4342 | 0.96200 |
| Species | 1 | 0.5139 | 0.51394 | 1.3625 | 0.24781 |
| Day | 2 | 1.5478 | 0.77390 | 2.0516 | 0.13760 |
| Drought : heat shock | 1 | 1.0394 | 1.03941 | 2.7555 | 0.10223 |
| Day : drought | 2 | 0.5054 | 0.25271 | 0.6699 | 0.51559 |
| Day : heat shock | 2 | 0.2370 | 0.11852 | 0.3142 | 0.73158 |
| Species : drought | 1 | 0.2658 | 0.26583 | 0.7047 | 0.40459 |
| Species : heat shock | 1 | 1.1777 | 1.17769 | 3.1221 | 0.08241 |
| Species : day | 2 | 0.5238 | 0.26192 | 0.6944 | 0.50343 |
| Day : drought : heat shock | 2 | 0.5747 | 0.28733 | 0.7617 | 0.47139 |
| Species : drought : heat shock | 1 | 0.0649 | 0.06492 | 0.1721 | 0.67975 |
| Species : day : drought | 2 | 0.8013 | 0.40064 | 1.0621 | 0.35225 |
| Speces : day : heat shock | 2 | 0.9065 | 0.45325 | 1.2016 | 0.30797 |
| Species : day : drought : heat shock | 2 | 0.3440 | 0.17198 | 0.4559 | 0.63609 |
| Error (split-plot) | 59 | 22.2557 | 0.37721 |  |  |

Supplementary Table 8

| **Factor** | **Df** | **Sum sq** | **Mean sq** | **F value** | **Pr(> F)** |
| --- | --- | --- | --- | --- | --- |
| Drought | 1 | 0.10204 | 0.102040 | 5.8941 | 0.02927* |
| Error (main-plot) | 14 | 0.24237 | 0.017312 |  |  |
| Heat shock | 1 | 0.2891 | 0.28908 | 2.0159 | 0.160918 |
| Block | 15 | 1.9510 | 0.13007 | 0.9071 | 0.560724 |
| Species | 1 | 0.6372 | 0.63723 | 4.4439 | 0.039284* |
| Day | 2 | 0.9462 | 0.47310 | 3.2993 | 0.038328* |
| Drought : heat shock | 1 | 0.0729 | 0.07290 | 0.5084 | 0.478639 |
| Day : drought | 2 | 0.3011 | 0.15057 | 1.0501 | 0.356362 |
| Day : heat shock | 2 | 0.0783 | 0.03914 | 0.2730 | 0.762082 |
| Species : drought | 1 | 0.1421 | 0.14212 | 0.9911 | 0.323533 |
| Species : heat shock | 1 | 0.1297 | 0.12974 | 0.9048 | 0.345386 |
| Species : day | 2 | 0.7073 | 0.35367 | 2.4664 | 0.093604 |
| Day : drought : heat shock | 2 | 1.0368 | 0.51839 | 3.6151 | 0.033036* |
| Species : drought : heat shock | 1 | 0.6732 | 0.67322 | 4.6949 | 0.034303* |
| Species : day : drought | 2 | 2.3969 | 1.19847 | 8.3578 | 0.000637* |
| Speces : day : heat shock | 2 | 0.5126 | 0.25629 | 1.7873 | 0.176362 |
| Species : day : drought : heat shock | 2 | 0.1205 | 0.06027 | 0.4203 | 0.658799 |
| Error (split-plot) | 59 | 8.4603 | 0.14340 |  |  |

Supplementary Table 9

| **Factor** | **Df** | **Sum sq** | **Mean sq** | **F value** | **Pr(> F)** |
| --- | --- | --- | --- | --- | --- |
| Drought | 1 | 0.000971 | 0.0009706 | 0.0751 | 0.788 |
| Error (main-plot) | 14 | 0.180877 | 0.0129198 |  |  |
| Heat shock | 1 | 0.3697 | 0.36967 | 1.2272 | 0.27245 |
| Block | 15 | 1.2975 | 0.08650 | 0.2871 | 0.99499 |
| Species | 1 | 0.1694 | 0.16939 | 0.5623 | 0.45630 |
| Day | 2 | 0.4506 | 0.22528 | 0.7479 | 0.47781 |
| Drought : heat shock | 1 | 0.4629 | 0.46290 | 1.5367 | 0.22002 |
| Day : drought | 2 | 0.1703 | 0.08514 | 0.2826 | 0.75481 |
| Day : heat shock | 2 | 0.0394 | 0.01971 | 0.0654 | 0.93672 |
| Species : drought | 1 | 0.0223 | 0.02225 | 0.0739 | 0.78672 |
| Species : heat shock | 1 | 1.7441 | 1.74410 | 5.7899 | 0.01927* |
| Species : day | 2 | 1.1308 | 0.56539 | 1.8769 | 0.16208 |
| Day : drought : heat shock | 2 | 0.4671 | 0.23353 | 0.7753 | 0.46521 |
| Species : drought : heat shock | 1 | 0.1913 | 0.19126 | 0.6349 | 0.42875 |
| Species : day : drought | 2 | 1.1827 | 0.59133 | 1.9631 | 0.14949 |
| Speces : day : heat shock | 2 | 0.1603 | 0.08013 | 0.2660 | 0.76735 |
| Species : day : drought : heat shock | 2 | 0.2171 | 0.10854 | 0.3603 | 0.69898 |
| Error (split-plot) | 59 | 17.7725 | 0.30123 |  |  |

Supplementary Table 10

| **Factor** | **Df** | **Sum sq** | **Mean sq** | **F value** | **Pr(> F)** |
| --- | --- | --- | --- | --- | --- |
| Drought | 1 | 3.5244 | 3.5244 | 5.1451 | 0.03966* |
| Error (main-plot) | 14 | 9.5900 | 0.6850 |  |  |
| Heat shock | 1 | 3.960 | 3.960 | 0.7669 | 0.384730 |
| Block | 15 | 66.756 | 4.450 | 0.8618 | 0.607878 |
| Species | 1 | 0.450 | 0.450 | 0.0872 | 0.768777 |
| Day | 2 | 59.359 | 29.680 | 5.7475 | 0.005242* |
| Drought : heat shock | 1 | 11.480 | 11.480 | 2.2231 | 0.141291 |
| Day : drought | 2 | 4.096 | 2.048 | 0.3966 | 0.674397 |
| Day : heat shock | 2 | 3.960 | 3.960 | 0.7669 | 0.384730 |
| Species : drought | 1 | 8.284 | 8.284 | 1.6043 | 0.210273 |
| Species : heat shock | 1 | 6.826 | 6.826 | 1.3218 | 0.254903 |
| Species : day | 2 | 7.207 | 3.603 | 0.6978 | 0.501732 |
| Day : drought : heat shock | 2 | 14.632 | 7.316 | 1.4168 | 0.250616 |
| Species : drought : heat shock | 1 | 7.855 | 7.855 | 1.5211 | 0.222340 |
| Species : day : drought | 2 | 64.485 | 32.243 | 6.2438 | 0.003470* |
| Speces : day : heat shock | 2 | 27.682 | 13.841 | 2.6803 | 0.076886 |
| Species : day : drought : heat shock | 2 | 2.850 | 1.425 | 0.2759 | 0.759847 |
| Error (split-plot) | 59 | 304.670 | 5.164 |  |  |

Supplementary Table 11

| **Factor** | **Df** | **Sum sq** | **Mean sq** | **F value** | **Pr(> F)** |
| --- | --- | --- | --- | --- | --- |
| Drought | 1 | 10.579 | 10.5792 | 5.1289 | 0.03993* |
| Error (main-plot) | 14 | 28.877 | 2.0627 |  |  |
| Heat shock | 1 | 63.53 | 63.53 | 1.6632 | 0.20221 |
| Block | 15 | 392.78 | 26.19 | 0.6856 | 0.78804 |
| Species | 1 | 1034.19 | 1034.19 | 27.0757 | 2.586e-06* |
| Day | 2 | 209.28 | 104.64 | 2.7395 | 0.07283 |
| Drought : heat shock | 1 | 2.79 | 2.79 | 0.0731 | 0.78777 |
| Day : drought | 2 | 160.79 | 80.39 | 2.1048 | 0.13093 |
| Day : heat shock | 2 | 61.55 | 30.77 | 0.8056 | 0.45165 |
| Species : drought | 1 | 14.54 | 14.54 | 0.3807 | 0.53961 |
| Species : heat shock | 1 | 0.00 | 0.00 | 0.0001 | 0.99303 |
| Species : day | 2 | 10.45 | 5.22 | 0.1367 | 0.87248 |
| Day : drought : heat shock | 2 | 34.94 | 17.47 | 0.4574 | 0.63515 |
| Species : drought : heat shock | 1 | 0.99 | 0.99 | 0.0259 | 0.87264 |
| Species : day : drought | 2 | 107.94 | 53.97 | 1.4130 | 0.25152 |
| Speces : day : heat shock | 2 | 13.18 | 6.59 | 0.1726 | 0.84191 |
| Species : day : drought : heat shock | 2 | 35.89 | 17.95 | 0.4698 | 0.62742 |
| Error (split-plot) | 59 | 2253.58 | 38.20 |  |  |
